# 6-Bromo-1-[2-(2-oxo-1,3-oxazolidin-3-yl)eth­yl]-1*H*-imidazo[4,5-*b*]pyridin-2(3*H*)-one

**DOI:** 10.1107/S1600536810002679

**Published:** 2010-01-27

**Authors:** H. Bel-Ghacham, Y. Kandri Rodi, Natalie Saffon, El Mokhtar Essassi, Seik Weng Ng

**Affiliations:** a Laboratoire de Chimie Organique Appliquée, Faculté des Sciences et Techniques, Université Sidi Mohamed Ben Abdallah, Fés, Morocco; b Service Commun Rayons-X FR2599, Université Paul Sabatier Bâtiment 2R1, 118 route de Narbonne, Toulouse, France; c Laboratoire de Chimie Organique Hétérocyclique, Pôle de Compétences Pharmacochimie, Université Mohammed V-Agdal, BP 1014 Avenue Ibn Batout, Rabat, Morocco; d Department of Chemistry, University of Malaya, 50603 Kuala Lumpur, Malaysia

## Abstract

The title compound, C_11_H_11_BrN_4_O_3_, features an ethane fragment substituted with an almost planar (r.m.s. deviation = 0.019 Å) imidazo[4,5-*b*]pyridone ring system and an envelope-shaped oxazolidine unit on separate C atoms. The N—CH_2_—CH_2_—N torsion angle is 52.5 (4)°. In the crystal, pairs of mol­ecules are linked by N—H⋯O hydrogen bonds into centrosymmetric dimers.

## Related literature

For the medicinal properties of imidazo[4,5-*b*]pyridines, see: Barraclough *et al.* (1990 ▶); Bianchi *et al.* (1983 ▶); Clark *et al.* (1978 ▶); Janssens *et al.* (1985 ▶); Temple *et al.* (1987 ▶).
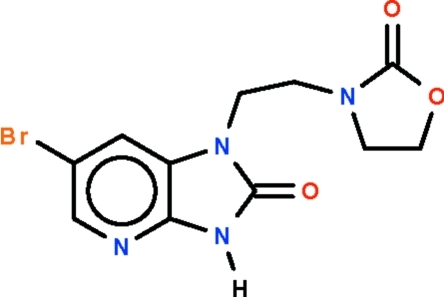


## Experimental

### 

#### Crystal data

C_11_H_11_BrN_4_O_3_
*M* *_r_* = 327.15
Monoclinic, 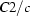

*a* = 27.0174 (11) Å
*b* = 6.0141 (2) Å
*c* = 16.6121 (6) Å
β = 110.343 (2)°
*V* = 2530.87 (16) Å^3^
*Z* = 8
Mo *K*α radiation
μ = 3.26 mm^−1^
*T* = 173 K
0.40 × 0.20 × 0.05 mm

#### Data collection

Bruker APEXII diffractometer
Absorption correction: multi-scan (*SADABS*; Sheldrick, 1996 ▶) *T*_min_ = 0.356, *T*_max_ = 0.854
9174 measured reflections
2224 independent reflections
1633 reflections with *I* > 2σ(*I*)
*R*_int_ = 0.062
Standard reflections: 0

#### Refinement

*R*[*F* ^2^ > 2σ(*F* ^2^)] = 0.038
*wR*(*F* ^2^) = 0.077
*S* = 1.02
2224 reflections
176 parameters
1 restraint
H atoms treated by a mixture of independent and constrained refinement
Δρ_max_ = 0.36 e Å^−3^
Δρ_min_ = −0.36 e Å^−3^

### 

Data collection: *APEX2* (Bruker, 2005 ▶); cell refinement: *SAINT* (Bruker, 2005 ▶); data reduction: *SAINT*; program(s) used to solve structure: *SHELXS97* (Sheldrick, 2008 ▶); program(s) used to refine structure: *SHELXL97* (Sheldrick, 2008 ▶); molecular graphics: *X-SEED* (Barbour, 2001 ▶); software used to prepare material for publication: *publCIF* (Westrip, 2010 ▶).

## Supplementary Material

Crystal structure: contains datablocks global, I. DOI: 10.1107/S1600536810002679/bt5180sup1.cif

Structure factors: contains datablocks I. DOI: 10.1107/S1600536810002679/bt5180Isup2.hkl

Additional supplementary materials:  crystallographic information; 3D view; checkCIF report

## Figures and Tables

**Table 1:** Hydrogen-bond geometry (Å, °)

*D*—H⋯*A*	*D*—H	H⋯*A*	*D*⋯*A*	*D*—H⋯*A*
N3—H3⋯O3^i^	0.86 (1)	1.94 (1)	2.781 (4)	167 (3)

Symmetry code: (i) 
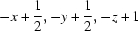
.

**Fig. 1.**
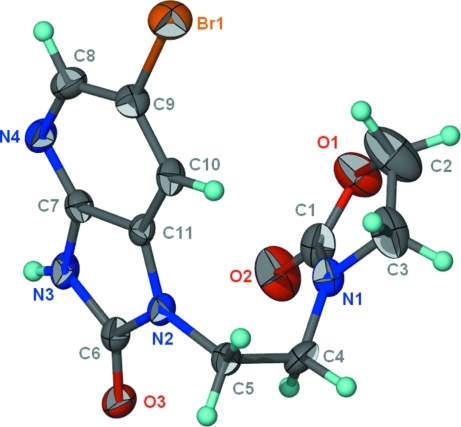
Anisotropic displacement ellipsoid plot (Barbour, 2001) of C11H11BrN4O3 at the 70% probability level; hydrogen atoms are drawn as spheres of an arbitrary radius.

## supplementary crystallographic information

### Experimental

To 6-bromo-1,3-dihydro-imidazo[4,5-*b*]pyridin-2-one (1 mmol), potassium carbonate (4 mmol), and tetra-*n*-butylammonium bromide (0.1 mmol) in DMF (30 ml) was added  bis(2-chloroethyl)amine hydrochloride (2.5 mmol). The mixture was heated for 48 h. After the completion of the reaction (as monitored by TLC), the inorganic material salt was filtered and the solvent was removed under reduced pressure. The residue was purified by column chromatography on silica gel by using (ethylacetate/hexane: 2/1) as eluent to furnish colorless crystals.

### Refinement

Carbon-bound H-atoms were placed in calculated positions (C—H 0.94-0.99 Å) and were included in the refinement in the riding model approximation, with *U*(H) set to 1.2*U*(C). The amino H-atom was located in a difference Fourier map, and was refined with a distance restraint of N–H 0.86±0.01 Å; its displacement parameter  was refined isotropically.

### Crystal data

**Table:**

C_11_H_11_BrN_4_O_3_	*F*(000) = 1312
*M**_r_* = 327.15	*D*_x_ = 1.717 Mg m^−^^3^
Monoclinic, *C*2/*c*	Mo *K*α radiation, λ = 0.71073 Å
Hall symbol: -C 2yc	Cell parameters from 1559 reflections
*a* = 27.0174 (11) Å	θ = 2.6–22.4°
*b* = 6.0141 (2) Å	µ = 3.26 mm^−^^1^
*c* = 16.6121 (6) Å	*T* = 173 K
β = 110.343 (2)°	Plate, colorless
*V* = 2530.87 (16) Å^3^	0.40 × 0.20 × 0.05 mm
*Z* = 8	

### Data collection

**Table:**

Bruker APEXII diffractometer	2224 independent reflections
Radiation source: fine-focus sealed tube	1633 reflections with *I* > 2σ(*I*)
graphite	*R*_int_ = 0.062
φ and ω scans	θ_max_ = 25.0°, θ_min_ = 2.6°
Absorption correction: multi-scan (*SADABS*; Sheldrick, 1996)	*h* = −26→32
*T*_min_ = 0.356, *T*_max_ = 0.854	*k* = −7→7
9174 measured reflections	*l* = −19→19

### Refinement

**Table:**

Refinement on *F*^2^	Primary atom site location: structure-invariant direct methods
Least-squares matrix: full	Secondary atom site location: difference Fourier map
*R*[*F*^2^ > 2σ(*F*^2^)] = 0.038	Hydrogen site location: inferred from neighbouring sites
*wR*(*F*^2^) = 0.077	H atoms treated by a mixture of independent and constrained refinement
*S* = 1.02	*w* = 1/[σ^2^(*F*_o_^2^) + (0.0269*P*)^2^ + 1.8498*P*] where *P* = (*F*_o_^2^ + 2*F*_c_^2^)/3
2224 reflections	(Δ/σ)_max_ = 0.001
176 parameters	Δρ_max_ = 0.36 e Å^−^^3^
1 restraint	Δρ_min_ = −0.36 e Å^−^^3^

### Fractional atomic coordinates and isotropic or equivalent isotropic displacement parameters (Å^2^)

**Table:**

	*x*	*y*	*z*	*U*_iso_*/*U*_eq_	
Br1	0.030317 (17)	1.14052 (7)	0.39869 (3)	0.03436 (15)	
O1	0.07133 (11)	0.4365 (4)	0.17338 (18)	0.0398 (8)	
O2	0.14847 (12)	0.2803 (5)	0.25167 (18)	0.0447 (8)	
O3	0.26482 (10)	0.4281 (4)	0.42343 (15)	0.0266 (6)	
N1	0.14287 (12)	0.6468 (5)	0.21101 (17)	0.0236 (7)	
N2	0.20224 (12)	0.7116 (5)	0.38942 (18)	0.0197 (7)	
N3	0.19871 (12)	0.4599 (5)	0.48386 (18)	0.0225 (7)	
H3	0.2052 (13)	0.339 (3)	0.5132 (18)	0.022 (10)*	
N4	0.12421 (12)	0.6066 (5)	0.51587 (18)	0.0242 (7)	
C1	0.12398 (18)	0.4418 (7)	0.2151 (2)	0.0307 (10)	
C2	0.05337 (18)	0.6589 (7)	0.1444 (3)	0.0565 (14)	
H2A	0.0354	0.7266	0.1812	0.068*	
H2B	0.0286	0.6568	0.0843	0.068*	
C3	0.10292 (17)	0.7865 (6)	0.1514 (3)	0.0366 (11)	
H3A	0.1079	0.7975	0.0953	0.044*	
H3B	0.1026	0.9376	0.1749	0.044*	
C4	0.19838 (15)	0.6905 (6)	0.2399 (2)	0.0261 (9)	
H4A	0.2065	0.7762	0.1951	0.031*	
H4B	0.2176	0.5474	0.2474	0.031*	
C5	0.21827 (15)	0.8193 (6)	0.3239 (2)	0.0221 (9)	
H5A	0.2573	0.8290	0.3439	0.027*	
H5B	0.2040	0.9725	0.3143	0.027*	
C6	0.22613 (15)	0.5212 (6)	0.4313 (2)	0.0220 (9)	
C7	0.15790 (14)	0.6070 (6)	0.4745 (2)	0.0198 (8)	
C8	0.08786 (15)	0.7701 (6)	0.4925 (2)	0.0268 (9)	
H8	0.0624	0.7777	0.5201	0.032*	
C9	0.08554 (15)	0.9282 (6)	0.4302 (2)	0.0227 (9)	
C10	0.12232 (15)	0.9304 (6)	0.3887 (2)	0.0220 (9)	
H10	0.1218	1.0386	0.3468	0.026*	
C11	0.15928 (14)	0.7649 (6)	0.4130 (2)	0.0183 (8)	

### Atomic displacement parameters (Å^2^)

**Table:**

	*U*^11^	*U*^22^	*U*^33^	*U*^12^	*U*^13^	*U*^23^
Br1	0.0348 (3)	0.0294 (3)	0.0412 (2)	0.0106 (2)	0.01603 (19)	0.0026 (2)
O1	0.0332 (19)	0.0294 (17)	0.0488 (19)	−0.0102 (14)	0.0042 (15)	−0.0006 (14)
O2	0.055 (2)	0.0231 (17)	0.0475 (19)	0.0033 (15)	0.0068 (16)	0.0151 (14)
O3	0.0277 (17)	0.0288 (16)	0.0264 (14)	0.0084 (13)	0.0134 (13)	0.0087 (11)
N1	0.029 (2)	0.0179 (18)	0.0216 (16)	−0.0027 (16)	0.0057 (14)	0.0021 (14)
N2	0.0232 (18)	0.0189 (17)	0.0178 (15)	−0.0005 (14)	0.0081 (14)	0.0051 (13)
N3	0.030 (2)	0.0186 (19)	0.0194 (17)	0.0056 (15)	0.0091 (15)	0.0093 (14)
N4	0.0270 (19)	0.0258 (19)	0.0222 (16)	0.0046 (15)	0.0116 (15)	0.0050 (14)
C1	0.042 (3)	0.024 (2)	0.026 (2)	−0.005 (2)	0.010 (2)	−0.0023 (18)
C2	0.043 (3)	0.035 (3)	0.072 (3)	0.003 (3)	−0.005 (3)	0.003 (3)
C3	0.047 (3)	0.021 (2)	0.033 (2)	0.006 (2)	0.003 (2)	0.0018 (18)
C4	0.031 (2)	0.029 (2)	0.025 (2)	−0.0018 (18)	0.0185 (19)	0.0000 (17)
C5	0.023 (2)	0.023 (2)	0.0200 (19)	−0.0004 (17)	0.0075 (16)	0.0040 (16)
C6	0.025 (2)	0.022 (2)	0.0183 (19)	−0.0003 (18)	0.0070 (18)	0.0022 (16)
C7	0.021 (2)	0.018 (2)	0.0183 (18)	0.0015 (17)	0.0048 (16)	0.0007 (16)
C8	0.026 (2)	0.031 (2)	0.027 (2)	−0.0008 (19)	0.0140 (19)	−0.0025 (18)
C9	0.024 (2)	0.019 (2)	0.025 (2)	0.0039 (16)	0.0088 (18)	−0.0012 (16)
C10	0.029 (2)	0.018 (2)	0.0182 (19)	0.0031 (17)	0.0066 (17)	0.0022 (15)
C11	0.022 (2)	0.019 (2)	0.0143 (18)	−0.0030 (17)	0.0064 (17)	−0.0032 (16)

### Geometric parameters (Å, °)

**Table:**

Br1—C9	1.894 (4)	C2—C3	1.512 (6)
O1—C1	1.349 (5)	C2—H2A	0.9900
O1—C2	1.447 (5)	C2—H2B	0.9900
O2—C1	1.213 (4)	C3—H3A	0.9900
O3—C6	1.232 (4)	C3—H3B	0.9900
N1—C1	1.345 (5)	C4—C5	1.521 (5)
N1—C4	1.431 (4)	C4—H4A	0.9900
N1—C3	1.452 (5)	C4—H4B	0.9900
N2—C6	1.378 (4)	C5—H5A	0.9900
N2—C11	1.385 (4)	C5—H5B	0.9900
N2—C5	1.457 (4)	C7—C11	1.405 (5)
N3—C6	1.377 (4)	C8—C9	1.390 (5)
N3—C7	1.380 (4)	C8—H8	0.9500
N3—H3	0.859 (10)	C9—C10	1.393 (5)
N4—C7	1.318 (4)	C10—C11	1.368 (5)
N4—C8	1.348 (4)	C10—H10	0.9500
			
C1—O1—C2	108.7 (3)	C5—C4—H4A	108.9
C1—N1—C4	121.6 (3)	N1—C4—H4B	108.9
C1—N1—C3	111.1 (3)	C5—C4—H4B	108.9
C4—N1—C3	124.5 (3)	H4A—C4—H4B	107.7
C6—N2—C11	109.9 (3)	N2—C5—C4	110.9 (3)
C6—N2—C5	122.6 (3)	N2—C5—H5A	109.5
C11—N2—C5	127.2 (3)	C4—C5—H5A	109.5
C6—N3—C7	110.0 (3)	N2—C5—H5B	109.5
C6—N3—H3	123 (2)	C4—C5—H5B	109.5
C7—N3—H3	127 (2)	H5A—C5—H5B	108.0
C7—N4—C8	114.5 (3)	O3—C6—N3	127.5 (3)
O2—C1—N1	127.5 (4)	O3—C6—N2	126.1 (3)
O2—C1—O1	122.2 (4)	N3—C6—N2	106.4 (3)
N1—C1—O1	110.3 (3)	N4—C7—N3	127.6 (3)
O1—C2—C3	104.9 (3)	N4—C7—C11	125.3 (3)
O1—C2—H2A	110.8	N3—C7—C11	107.1 (3)
C3—C2—H2A	110.8	N4—C8—C9	123.6 (3)
O1—C2—H2B	110.8	N4—C8—H8	118.2
C3—C2—H2B	110.8	C9—C8—H8	118.2
H2A—C2—H2B	108.8	C8—C9—C10	121.3 (3)
N1—C3—C2	100.8 (3)	C8—C9—Br1	119.6 (3)
N1—C3—H3A	111.6	C10—C9—Br1	119.1 (3)
C2—C3—H3A	111.6	C11—C10—C9	114.9 (3)
N1—C3—H3B	111.6	C11—C10—H10	122.6
C2—C3—H3B	111.6	C9—C10—H10	122.6
H3A—C3—H3B	109.4	C10—C11—N2	133.1 (3)
N1—C4—C5	113.3 (3)	C10—C11—C7	120.3 (3)
N1—C4—H4A	108.9	N2—C11—C7	106.6 (3)
			
C4—N1—C1—O2	10.3 (6)	C5—N2—C6—N3	−176.1 (3)
C3—N1—C1—O2	171.9 (4)	C8—N4—C7—N3	−179.2 (3)
C4—N1—C1—O1	−171.3 (3)	C8—N4—C7—C11	2.5 (5)
C3—N1—C1—O1	−9.7 (4)	C6—N3—C7—N4	−177.8 (3)
C2—O1—C1—O2	174.3 (4)	C6—N3—C7—C11	0.8 (4)
C2—O1—C1—N1	−4.2 (4)	C7—N4—C8—C9	−0.2 (5)
C1—O1—C2—C3	15.5 (5)	N4—C8—C9—C10	−1.6 (6)
C1—N1—C3—C2	18.3 (4)	N4—C8—C9—Br1	176.7 (3)
C4—N1—C3—C2	179.3 (3)	C8—C9—C10—C11	1.2 (5)
O1—C2—C3—N1	−19.6 (4)	Br1—C9—C10—C11	−177.2 (3)
C1—N1—C4—C5	−107.3 (4)	C9—C10—C11—N2	−178.5 (4)
C3—N1—C4—C5	93.6 (4)	C9—C10—C11—C7	0.9 (5)
C6—N2—C5—C4	75.4 (4)	C6—N2—C11—C10	−178.0 (4)
C11—N2—C5—C4	−97.5 (4)	C5—N2—C11—C10	−4.3 (6)
N1—C4—C5—N2	52.5 (4)	C6—N2—C11—C7	2.6 (4)
C7—N3—C6—O3	179.9 (4)	C5—N2—C11—C7	176.2 (3)
C7—N3—C6—N2	0.8 (4)	N4—C7—C11—C10	−2.9 (6)
C11—N2—C6—O3	178.8 (4)	N3—C7—C11—C10	178.5 (3)
C5—N2—C6—O3	4.8 (6)	N4—C7—C11—N2	176.6 (3)
C11—N2—C6—N3	−2.1 (4)	N3—C7—C11—N2	−2.0 (4)

### Hydrogen-bond geometry (Å, °)

**Table:**

*D*—H···*A*	*D*—H	H···*A*	*D*···*A*	*D*—H···*A*
N3—H3···O3^i^	0.86 (1)	1.94 (1)	2.781 (4)	167 (3)

Symmetry codes: (i) −*x*+1/2, −*y*+1/2, −*z*+1.
